# Geometric inequalities for warped product bi-slant submanifolds with a warping function

**DOI:** 10.1186/s13660-018-1843-3

**Published:** 2018-09-27

**Authors:** Aliya Naaz Siddiqui, Mohammad Hasan Shahid, Jae Won Lee

**Affiliations:** 1 Department of Mathematics, Faculty of Natural Sciences, Jamia Millia Islamia, New Delhi, India; 2 Department of Mathematics Education and RINS, Gyeongsang National University, Jinju, South Korea

**Keywords:** 53C40, 53C42, 53C15, Nearly trans-Sasakian manifolds, Bi-slant submanifolds, Warped product bi-slant submanifolds

## Abstract

In this paper, we prove that the squared norm of the second fundamental form for bi-slant submanifolds with any codimension of nearly trans-Sasakian manifolds is bounded below by the gradient of a warping function and also find the conditions on which the equality holds. Some related examples are also provided.

## Introduction

In 1969, the idea of warped product manifolds was initiated by R.L. Bishop and B. O’Neil [1] with manifolds of negative curvature. These manifolds are natural generalizations of Riemannian product manifolds. This is an extremely interesting and innovative research topic for geometers. Several papers are available in literature. Many significant physical applications of warped product manifolds have been found (for example [2, 3]). Geometers are attracted to work on warped product manifolds. On the other hand, B.-Y. Chen [4] has introduced the notion of a CR-warped product submanifold in a Kaehler manifold and established a general inequality for a CR-warped product submanifold in the same ambient manifold. He also has discussed the classification of CR-warped products in complex Euclidean [4], complex projective and complex hyperbolic spaces [5] which satisfy the equality case of the derived inequality. Moreover, he has established many geometric inequalities for the second fundamental form for different warped product submanifolds of different ambient in terms of a warping function. Inspired by his work, many distinguished geometers have studied and obtained several sharp inequalities for warped product submanifolds in almost Hermitian manifolds and almost contact metric manifolds (see the monograph [6] and the references therein).

Our work is outlined as follows: In Sect. 2, we review some basic concepts and address the study of bi-slant submanifolds of nearly trans-Sasakian manifolds. In Sect. 3, we prepare lemmas to use in proving the main result of this paper. In Sect. 4, we define an orthonormal frame for warped product bi-slant submanifolds of an arbitrary nearly trans-Sasakian manifold and then we establish a sharp inequality for the second fundamental form in terms of a warping function. The equality case is also discussed. Finally, in Sect. 5, we investigate the triviality of warped product bi-slant submanifolds in nearly trans-Sasakian manifolds and some non-trivial examples are also provided.

## Nearly trans-Sasakian manifolds and their submanifolds

An odd dimensional smooth manifold $\overline{\mathcal{M}}$ has an almost contact metric structure $(\varphi, \xi, \upsilon, g)$ if there exist on $\overline{\mathcal{M}}$ a tensor field *φ* of type $(1, 1)$, a structure vector field *ξ*, a dual 1-form *υ*, and a Riemannian metric *g* such that [7]

1 $$\begin{aligned} \left . \begin{aligned} &\varphi^{2} = -I + \upsilon \otimes \xi, \qquad \varphi \circ \xi = 0, \qquad \upsilon(\xi) = 1, \qquad \upsilon(\mathcal{X}) = g(\mathcal{X}, \xi), \\ &g(\varphi \mathcal{X}, \mathcal{Y}) = -g(\mathcal{X}, \varphi \mathcal{Y}), \qquad g(\varphi \mathcal{X}, \varphi \mathcal{Y}) = g(\mathcal{X}, \mathcal{Y}) - \upsilon(\mathcal{X})\upsilon(\mathcal{Y}), \end{aligned} \right\} \end{aligned}$$

for any $\mathcal{X}, \mathcal{Y} \in \Gamma(T\overline{\mathcal{M}})$. An almost contact metric structure $(\varphi, \xi, \upsilon, g)$ on $\overline{\mathcal{M}}$ is called a *nearly trans-Sasakian structure* [8] if

2 $$ \begin{aligned}[b] (\overline{\nabla}_{\mathcal{X}} \varphi) \mathcal{Y} + (\overline{\nabla}_{\mathcal{Y}} \varphi)\mathcal{X} ={}& \lambda \bigl(2g(\mathcal{X}, \mathcal{Y})\xi - \upsilon(\mathcal{Y})\mathcal{X} - \upsilon(\mathcal{X})\mathcal{Y} \bigr) \\ &{}- \mu \bigl(\upsilon(\mathcal{Y})\varphi \mathcal{X} + \upsilon(\mathcal{X}) \varphi \mathcal{Y} \bigr) \end{aligned} $$

for some smooth functions *λ* and *μ* on $\overline{\mathcal{M}}$, and we say that the nearly trans-Sasakian structure is of type $(\lambda, \mu)$. The covariant derivative of the tensor field *φ* is given by

3 $$ (\overline{\nabla}_{\mathcal{X}}\varphi)\mathcal{Y} = \overline{ \nabla}_{\mathcal{X}}\varphi \mathcal{Y} - \varphi\overline{\nabla}_{\mathcal{X}} \mathcal{Y} $$

for any $\mathcal{X}, \mathcal{Y} \in \Gamma(T\overline{\mathcal{M}})$.

### Remark 1

A nearly trans-Sasakian structure of type $(\lambda, \mu)$ is

(i) *nearly Sasakian* [9], if $\lambda = 1, \mu = 0$;
(ii) *nearly Kenmotsu* [10], if $\lambda = 0, \mu = 1$;
(iii) *nearly cosymplectic* [11], if $\lambda = \mu = 0$.

Every Kenmotsu manifold is a nearly Kenmotsu manifold but the converse is not true. Also, a nearly Kenmotsu manifold is not a Sasakian manifold. On the other hand, every nearly Sasakian manifold with dimension greater than five is a Sasakian manifold.

We consider a Riemannian submanifold $\mathcal{M}$ isometrically immersed in an odd dimensional almost contact metric manifold $(\overline{\mathcal{M}}, \varphi, \xi, \upsilon, g)$ with induced metric *g*. We denote the Lie algebra of vector fields in $\mathcal{M}$ and the set of all vector fields normal to $\mathcal{M}$ by $\Gamma(T\mathcal{M})$ and $\Gamma(T^{\perp}\mathcal{M})$, respectively. Let ∇̅ be the Levi–Civita connection on $\overline{\mathcal{M}}$ and ∇ be the induced connection on $\mathcal{M}$. Then the Gauss and Weingarten formulae are respectively given below [12]:

$$\overline{\nabla}_{\mathcal{X}} \mathcal{Y} = \nabla_{\mathcal{X}}\mathcal{Y} + \zeta(\mathcal{X}, \mathcal{Y}), \qquad \overline{\nabla}_{\mathcal{X}}\mathcal{V} = - \Lambda_{\mathcal{V}}(\mathcal{X}) + \nabla_{\mathcal{X}}^{\perp} \mathcal{Y}, $$

for any $\mathcal{X}, \mathcal{Y} \in \Gamma(T\mathcal{M})$ and $\mathcal{V} \in \Gamma(T^{\perp}\mathcal{M})$. Here *ζ* and Λ are the bilinear symmetric second fundamental form of $\mathcal{M}$ in $\overline{\mathcal{M}}$ and the shape operator of $\mathcal{M}$, respectively. Both are related as $g(\zeta(\mathcal{X}, \mathcal{Y}), \mathcal{V})= g(\Lambda_{\mathcal{V}}(\mathcal{X}), \mathcal{Y})$ for any $\mathcal{X}, \mathcal{Y} \in \Gamma(T\mathcal{M})$ and $\mathcal{V} \in \Gamma(T^{\perp}\mathcal{M})$.

We assume that $\operatorname{dim}(\mathcal{M}) = m$ and $\operatorname{dim}(\overline{\mathcal{M}}) = 2n + 1$. Let $\{\mathcal{E}_{1}, \dots, \mathcal{E}_{m}\}$ be a local orthonormal frame of $T_{\wp}\mathcal{M}$ and $\{\mathcal{E}_{m+1}, \dots, \mathcal{E}_{2n + 1}\}$ be a local orthonormal frame of $T_{\wp}^{\perp}\mathcal{M}$, $\wp \in \mathcal{M}$. Then the mean curvature vector $\mathcal{H}$ of a submanifold $\mathcal{M}$ at ℘ is given by $\mathcal{H} = \frac{1}{m}\sum_{i=1}^{m}\zeta(\mathcal{E}_{i}, \mathcal{E}_{i})$. Also, we set $\zeta_{ij}^{r} = g(\zeta(\mathcal{E}_{i}, \mathcal{E}_{j}), \mathcal{E}_{r})$, $i,j \in\ \{1, \dots, m\}$, $r \in \{m+1, \dots, 2n+1\}$, and $\|\zeta\|^{2} = \sum_{i,j=1}^{m}g(\zeta(\mathcal{E}_{i}, \mathcal{E}_{j}), \zeta(\mathcal{E}_{i}, \mathcal{E}_{j}))$.

### Definition 1

([12])

A submanifold $\mathcal{M}$ of $\overline{\mathcal{M}}$ is said to be

(i) *totally umbilical* if its second fundamental form satisfies $\zeta(\mathcal{X}, \mathcal{Y})= g(\mathcal{X}, \mathcal{Y})\mathcal{H}$ for any $\mathcal{X}, \mathcal{Y} \in \Gamma(T\mathcal{M})$, where $\mathcal{H}$ is the *mean curvature vector* of $\mathcal{M}$ in $\overline{\mathcal{M}}$;
(ii) *totally geodesic* if $\zeta(\mathcal{X}, \mathcal{Y})= 0$ for any $\mathcal{X}, \mathcal{Y} \in \Gamma(T\mathcal{M})$;
(iii) *minimal* if $\mathcal{H} = 0$.

For any $\mathcal{X} \in \Gamma(T\mathcal{M})$ and $\mathcal{V} \in \Gamma(T^{\perp}\mathcal{M})$, respectively, we put $\varphi \mathcal{X} = \mathcal{P}\mathcal{X} + \mathcal{F}\mathcal{X}$ and $\varphi \mathcal{V} = B\mathcal{V} + C\mathcal{V}$, where $\mathcal{P}\mathcal{X}$ and $\mathcal{F}\mathcal{X}$ are the tangential and the normal components of $\varphi \mathcal{X}$, respectively. Similarly, $B\mathcal{V}$ and $C\mathcal{V}$ are the tangential and the normal components of $\varphi \mathcal{V}$, respectively. For their geometric relations, see [12].

### Definition 2

A submanifold $\mathcal{M}$ of an almost contact metric manifold $(\overline{\mathcal{M}}, \varphi, \xi, \upsilon, g)$ is said to be *invariant* if $\mathcal{F} \equiv 0$, that is, $\varphi \mathcal{X} \in \Gamma(T\mathcal{M})$, and *anti-invariant* if $\mathcal{P} \equiv 0$, that is, $\varphi \mathcal{X} \in \Gamma(T^{\perp}\mathcal{M})$ for any $\mathcal{X} \in \Gamma(T\mathcal{M})$.

### Definition 3

([13])

Let $\mathcal{M}$ be a submanifold of an almost contact metric manifold $(\overline{\mathcal{M}}, \varphi, \xi, \upsilon, g)$. If, for each non-zero vector $\mathcal{X} \in T_{\wp}\mathcal{M} - \{\xi_{\wp}\}$ and $\wp \in \mathcal{M}$, the angle $\vartheta(\mathcal{X})$ between $\varphi \mathcal{X}$ and $T_{\wp}\mathcal{M}$ is constant, then $\mathcal{M}$ is called a *slant submanifold*, and *ϑ* is called the *slant angle* of $\mathcal{M}$.

For slant submanifolds, the following facts are known [14]:

4 $$\begin{aligned} \left . \begin{aligned} &\mathcal{P}^{2}(\mathcal{X}) = \cos^{2}\vartheta\bigl(- \mathcal{X} + \upsilon(\mathcal{X})\xi\bigr), \\ &g(\mathcal{P}\mathcal{X}, \mathcal{P}\mathcal{Y}) = \cos^{2}\vartheta \bigl(g(\mathcal{X}, \mathcal{Y}) - \upsilon(\mathcal{Y})\upsilon(\mathcal{X}) \bigr), \\ &g(\mathcal{F}\mathcal{X}, \mathcal{F}\mathcal{Y}) = \sin^{2}\vartheta \bigl(g(\mathcal{X}, \mathcal{Y}) - \upsilon(\mathcal{Y})\upsilon(\mathcal{X}) \bigr), \end{aligned} \right\} \end{aligned}$$

for any $\mathcal{X}, \mathcal{Y} \in \Gamma(T\mathcal{M})$, where *ϑ* is the slant angle of $\mathcal{M}$.

There are some other important classes of submanifolds which are determined by the behavior of tangent bundle of the submanifold under the action of *φ* of $\overline{\mathcal{M}}$. A submanifold $\mathcal{M}$ of $\overline{\mathcal{M}}$ is called [15, 16]:

(i) *a contact CR-submanifold* of $\overline{\mathcal{M}}$ if there exists a differentiable distribution $\mathcal{D} : \wp \rightarrow \mathcal{D}_{p} \subset T_{\wp}\mathcal{M}$ such that $\mathcal{D}$ is invariant with respect to *φ* and the orthogonal complementary distribution $\mathcal{D}_{\perp}$ is anti-invariant with respect to *φ*. The tangent bundle $T\mathcal{M}$ has the orthogonal decomposition $T\mathcal{M} = \mathcal{D} \oplus \mathcal{D}_{\perp} \oplus \{\xi\}$, where $\{\xi\}$ is a 1-dimensional distribution which is spanned by *ξ*.
(ii) *a semi-slant submanifold* of $\overline{\mathcal{M}}$ if there exists a pair of orthogonal distributions $\mathcal{D}$ and $\mathcal{D}_{\vartheta}$ such that $T\mathcal{M} = \mathcal{D} \oplus \mathcal{D}_{\vartheta} \oplus \{\xi\}$, where $\mathcal{D}$ is invariant with respect to *φ* and $\mathcal{D}_{\vartheta}$ is proper slant.
(iii) *a pseudo-slant submanifold* of $\overline{\mathcal{M}}$ if there exists a pair of orthogonal distributions $\mathcal{D}_{\perp}$ and $\mathcal{D}_{\vartheta}$ such that $T\mathcal{M} = \mathcal{D}_{\perp} \oplus \mathcal{D}_{\vartheta} \oplus \{\xi\}$, where $\mathcal{D}_{\perp}$ is anti-invariant with respect to *φ* and $\mathcal{D}_{\vartheta}$ is proper slant.

### Definition 4

([17])

A submanifold $\mathcal{M}$ of an almost contact metric manifold $(\overline{\mathcal{M}}, \varphi, \xi, \upsilon, g)$ is said to be a *bi-slant submanifold* if there exists a pair of orthogonal distributions $\mathcal{D}_{\vartheta_{1}}$ and $\mathcal{D}_{\vartheta_{2}}$ on $\mathcal{M}$ such that

(i) The tangent space $T\mathcal{M}$ admits the orthogonal direct decomposition $T\mathcal{M} = \mathcal{D}_{\vartheta_{1}} \oplus \mathcal{D}_{\vartheta_{2}} \oplus \{\xi\}$;
(ii) $\mathcal{P}\mathcal{D}_{\vartheta_{1}} \perp \mathcal{D}_{\vartheta_{2}}$ and $\mathcal{P}\mathcal{D}_{\vartheta_{2}} \perp \mathcal{D}_{\vartheta_{1}}$;
(iii) Each distribution $\mathcal{D}_{\vartheta_{i}}$ is slant with slant angle $\vartheta_{i}$ for $i = 1, 2$.

### Remark 2

A bi-slant submanifold $\mathcal{M}$ is called *proper* if its bi-slant angles $\vartheta_{i} \neq 0, \frac{\pi}{2}$, for $i = 1, 2$. Otherwise,

(i) when $\vartheta_{1} = 0$ and $\vartheta_{2} = \frac{\pi}{2}$, then $\mathcal{M}$ is a *CR-submanifold*;
(ii) when $\vartheta_{1} = 0$ and $\vartheta_{2} \neq 0, \frac{\pi}{2}$, then $\mathcal{M}$ is a *semi-slant submanifold*;
(iii) when $\vartheta_{1} = \frac{\pi}{2}$ and $\vartheta_{2} \neq 0, \frac{\pi}{2}$, then $\mathcal{M}$ is a *pseudo-slant submanifold*.

For a bi-slant submanifold $\mathcal{M}$ of an almost contact metric manifold $(\overline{\mathcal{M}}, \varphi, \xi, \upsilon, g)$, the normal bundle $T^{\perp}\mathcal{M}$ is decomposed as

5 $$\begin{aligned} T^{\perp}\mathcal{M} = \mathcal{F}\mathcal{D}_{\vartheta_{1}} \oplus \mathcal{F}\mathcal{D}_{\vartheta_{2}} \oplus \nu, \end{aligned}$$

where *ν* is a *φ*-invariant normal subbundle of $\mathcal{M}$.

## Warped product bi-slant submanifolds

### Definition 5

([1])

Let $(\mathcal{M}_{1}, g_{1})$ and $(\mathcal{M}_{2}, g_{2})$ be two Riemannian manifolds and *h* be a positive differentiable function on $\mathcal{M}_{1}$. Let $\varrho: \mathcal{M}_{1} \times \mathcal{M}_{2} \longrightarrow \mathcal{M}_{1}$ and $\sigma: \mathcal{M}_{1} \times \mathcal{M}_{2} \longrightarrow \mathcal{M}_{2}$ be the canonical projection maps on $\mathcal{M}_{1} \times \mathcal{M}_{2}$, defined by $\varrho(\wp, \mathfrak{r}) = \wp$ and $\sigma(\wp, \mathfrak{r}) = \mathfrak{r}$ for any $(\wp, \mathfrak{r}) \in \mathcal{M}_{1} \times \mathcal{M}_{2}$. Then the warped product $\mathcal{M} = \mathcal{M}_{1} \times_{h} \mathcal{M}_{2}$ is the product manifold $\mathcal{M}_{1} \times \mathcal{M}_{2}$ equipped with the Riemannian structure such that $g(\mathcal{X}, \mathcal{Y}) = g_{1}(\varrho_{*} \mathcal{X}, \varrho_{*} \mathcal{Y}) + (h \circ \varrho)^{2} g_{2}(\sigma_{*} \mathcal{X}, \sigma_{*} \mathcal{Y})$ for any $\mathcal{X}, \mathcal{Y} \in \Gamma(T\mathcal{M})$, where ∗ is the symbol for the tangent maps. The function *h* is called a *warping function* of $\mathcal{M}$.

### Remark 3

([1, 5])

A warped product manifold is said to be *trivial* if its warping function is constant. In this case, the warped product manifold is a Riemannian product manifold. For the trivial warped product manifold $\mathcal{M} = \mathcal{M}_{1} \times_{h} \mathcal{M}_{2}$, submanifolds $\mathcal{M}_{1}$ and $\mathcal{M}_{2}$ are totally geodesic and totally umbilical of $\mathcal{M}$, respectively.

Here we define the notion of warped product bi-slant submanifolds of a nearly trans-Sasakian manifold:

### Definition 6

A warped product $\mathcal{M}_{1} \times_{h} \mathcal{M}_{2}$ of two slant submanifolds $\mathcal{M}_{1}$ and $\mathcal{M}_{2}$ with slant angles $\vartheta_{1}$ and $\vartheta_{2}$, respectively, of a nearly trans-Sasakian manifold $(\overline{\mathcal{M}}, \varphi, \xi, \upsilon, g)$ is called a *warped product bi-slant submanifold*.

Now, we recall the following general result for warped product manifolds:

### Lemma 1

([1])

*For a warped product manifold* $\mathcal{M} = \mathcal{M}_{1} \times_{h} \mathcal{M}_{2}$ *with a warping function* *h*, *the following formulas hold*:

(i) $\nabla_{\mathcal{X}}\mathcal{Y} \in T\mathcal{M}_{1}$ *is the lift of* $\nabla_{\mathcal{X}}\mathcal{Y}$ *on* $\mathcal{M}_{1}$,
(ii) $\nabla_{\mathcal{X}}\mathcal{Z} = \nabla_{\mathcal{Z}}\mathcal{X} = (\mathcal{X} \ln h)\mathcal{Z}$,
(iii) $\nabla_{\mathcal{Z}}\mathcal{W} = \nabla_{\mathcal{Z}}^{\mathcal{M}_{2}}\mathcal{W} - g(\mathcal{Z}, \mathcal{W})\nabla \ln h$,

*for any* $\mathcal{X}, \mathcal{Y} \in \Gamma(T\mathcal{M}_{1})$ *and* $\mathcal{Z}, \mathcal{W} \in \Gamma(T\mathcal{M}_{2})$, *where* ∇ *and* $\nabla^{\mathcal{M}_{2}}$ *are the Levi–Civita connections on* $\mathcal{M}$ *and* $\mathcal{M}_{2}$, *respectively*.

For a differentiable function *h* on a Riemannian manifold $\mathcal{M}$ of dimension *n*, the gradient of *h*, ∇*h*, is defined by

6 $$\begin{aligned} g(\nabla h, \mathcal{X}) = \mathcal{X}h, \end{aligned}$$

for any $\mathcal{X} \in \Gamma(T\mathcal{M})$. As a consequence, we have $\|\nabla h\|^{2} = \sum_{i = 1}^{n}(\mathcal{E}_{i}(h))^{2}$ for a local orthonormal frame $\{\mathcal{E}_{1}, \dots, \mathcal{E}_{n}\}$ on $\mathcal{M}$.

We consider the warped product bi-slant submanifold $\mathcal{M} = \mathcal{M}_{1} \times_{h} \mathcal{M}_{2}$ of a nearly trans-Sasakian manifold $(\overline{\mathcal{M}}, \varphi, \xi, \upsilon, g)$ such that the structure vector field *ξ* is tangent to $\mathcal{M}_{1}$. The following lemma plays a crucial role in our main result.

### Lemma 2

*Let* $\mathcal{M} = \mathcal{M}_{1} \times_{h} \mathcal{M}_{2}$ *be a warped product bi*-*slant submanifold of a nearly trans*-*Sasakian manifold* $(\overline{\mathcal{M}}, \varphi, \xi, \upsilon, g)$. *Then*

(i) $(\xi \ln h) = \mu$,
(ii) $g(\zeta(\mathcal{X}, \mathcal{Z}), \mathcal{F}\mathcal{Z}) = g(\zeta(\mathcal{Z}, \mathcal{Z}), \mathcal{F}\mathcal{X}) - [\lambda \upsilon(\mathcal{X}) + (\mathcal{P}\mathcal{X} \ln h) ] \|\mathcal{Z}\|^{2}$,
(iii) $g(\zeta(\mathcal{X}, \mathcal{P}\mathcal{Z}), \mathcal{F}\mathcal{P}\mathcal{Z}) = g(\zeta(\mathcal{P}\mathcal{Z}, \mathcal{P}\mathcal{Z}), \mathcal{F}\mathcal{X}) - [\lambda \upsilon(\mathcal{X}) + (\mathcal{P}\mathcal{X} \ln h) ] \cos^{2}\vartheta_{2} \|\mathcal{Z}\|^{2}$,
(iv) $g(\zeta(\mathcal{P}\mathcal{X}, \mathcal{Z}), \mathcal{F}\mathcal{Z}) = g(\zeta(\mathcal{Z}, \mathcal{Z}), \mathcal{F}\mathcal{P}\mathcal{X}) + [(\mathcal{X} \ln h) \cos^{2}\vartheta_{1} - \mu \upsilon(\mathcal{X}) \cos^{2}\vartheta_{1} ] \|\mathcal{Z}\|^{2}$,
(v) $g(\zeta(\mathcal{P}\mathcal{X}, \mathcal{P}\mathcal{Z}), \mathcal{F}\mathcal{P}\mathcal{Z}) = g(\zeta(\mathcal{P}\mathcal{Z}, \mathcal{P}\mathcal{Z}), \mathcal{F}\mathcal{P}\mathcal{X}) + [(\mathcal{X} \ln h) \cos^{2}\vartheta_{1} - \mu \upsilon(\mathcal{X}) \cos^{2}\vartheta_{1} ] \cos^{2}\vartheta_{2} \|\mathcal{Z}\|^{2}$,
(vi) $g(\zeta(\mathcal{X}, \mathcal{P}\mathcal{Z}), \mathcal{F}\mathcal{Z}) = - g(\zeta(\mathcal{X}, \mathcal{Z}), \mathcal{F}\mathcal{P}\mathcal{Z}) = \frac{1}{3} [- (\mathcal{X} \ln h) + \mu \upsilon(\mathcal{X}) ] \cos^{2}\vartheta_{2} \|\mathcal{Z}\|^{2}$,

*for* $\mathcal{X} \in \Gamma(T\mathcal{M}_{1})$ *and* $\mathcal{Z} \in \Gamma(T\mathcal{M}_{2})$.

### Proof

The proof of assertion (i) follows from Lemma 3.1(i) in [18]. In order to prove assertion (ii), we consider

$$\begin{aligned} g\bigl(\zeta(\mathcal{X}, \mathcal{Z}), \mathcal{F}\mathcal{Z}\bigr) = g( \overline{\nabla}_{\mathcal{Z}}\mathcal{X}, \varphi \mathcal{Z} - \mathcal{P} \mathcal{Z}) = g(\overline{\nabla}_{\mathcal{Z}}\mathcal{X}, \varphi \mathcal{Z}) - g(\overline{\nabla}_{\mathcal{Z}}\mathcal{X}, \mathcal{P}\mathcal{Z}) \end{aligned}$$

for $\mathcal{X}, \mathcal{Y} \in \Gamma(T\mathcal{M}_{1})$ and $\mathcal{Z} \in \Gamma(T\mathcal{M}_{2})$. From (2) and Lemma 1(ii), we derive

$$\begin{aligned} g\bigl(\zeta(\mathcal{X}, \mathcal{Z}), \mathcal{F}\mathcal{Z}\bigr) =& - g \bigl(\mathcal{X}, (\overline{\nabla}_{\mathcal{Z}}\varphi)\mathcal{Z}\bigr) - g( \mathcal{X}, \varphi \overline{\nabla}_{\mathcal{Z}}\mathcal{Z}) \\ =& - \lambda \upsilon(\mathcal{X}) \Vert \mathcal{Z} \Vert ^{2} - (\mathcal{P}\mathcal{X} \ln h) \Vert \mathcal{Z} \Vert ^{2} + g \bigl(\zeta(\mathcal{Z}, \mathcal{Z}), \mathcal{F}\mathcal{X}\bigr), \end{aligned}$$

or

$$\begin{aligned} g\bigl(\zeta(\mathcal{X}, \mathcal{Z}), \mathcal{F} \mathcal{Z}\bigr) =& g\bigl(\zeta(\mathcal{Z}, \mathcal{Z}), \mathcal{F} \mathcal{X}\bigr) - \bigl[\lambda \upsilon(\mathcal{X}) + (\mathcal{P}\mathcal{X} \ln h) \bigr] \Vert \mathcal{Z} \Vert ^{2}. \end{aligned}$$

This is assertion (ii). If we replace $\mathcal{Z}$ by $\mathcal{P}\mathcal{Z}$ for $\mathcal{Z} \in \Gamma(T\mathcal{M}_{2})$ and $\mathcal{X}$ by $\mathcal{P}\mathcal{X}$ for $\mathcal{X} \in \Gamma(T\mathcal{M}_{1})$ in assertion (ii), we can easily get assertions (iii) and (iv). Thus, assertion (v) can be obtained by replacing $\mathcal{Z}$ by $\mathcal{P}\mathcal{Z}$ for $\mathcal{Z} \in \Gamma(T\mathcal{M}_{2})$ and $\mathcal{X}$ by $\mathcal{P}\mathcal{X}$ for $\mathcal{X} \in \Gamma(T\mathcal{M}_{1})$ simultaneously in (ii). Now, consider for $\mathcal{X}, \mathcal{Y} \in \Gamma(T\mathcal{M}_{1})$ and $\mathcal{Z} \in \Gamma(T\mathcal{M}_{2})$ we have

$$\begin{aligned} g\bigl(\zeta(\mathcal{X}, \mathcal{P}\mathcal{Z}), \mathcal{F}\mathcal{Z}\bigr) =& - g(\mathcal{P}\mathcal{Z}, \overline{\nabla}_{\mathcal{X}} \mathcal{F} \mathcal{Z}) = - g(\mathcal{P}\mathcal{Z}, \overline{\nabla}_{\mathcal{X}}\varphi \mathcal{Z}) + g(\overline{\nabla}_{\mathcal{X}}\mathcal{P}\mathcal{Z}, \mathcal{P}\mathcal{Z}). \end{aligned}$$

From (3), (2), and Lemma 1$(ii)$, we obtain

7 $$ \begin{aligned}[b] g\bigl(\zeta(\mathcal{X}, \mathcal{P}\mathcal{Z}), \mathcal{F}\mathcal{Z}\bigr) ={}& - g(\mathcal{P}\mathcal{Z}, \varphi \overline{ \nabla}_{\mathcal{X}}\mathcal{Z}) - g\bigl(\mathcal{P}\mathcal{Z}, (\overline{ \nabla}_{\mathcal{X}}\varphi)\mathcal{Z}\bigr) \\ &{} + (\mathcal{X} \ln h) \cos^{2}\vartheta_{2} \Vert \mathcal{Z} \Vert ^{2} \\ ={}& - g\bigl(\mathcal{P}\mathcal{Z}, (\overline{\nabla}_{\mathcal{X}}\varphi) \mathcal{Z}\bigr) + g\bigl(\zeta(\mathcal{X}, \mathcal{Z})\mathcal{FP}\mathcal{Z} \bigr) \\ ={}& g\bigl(\mathcal{P}\mathcal{Z}, (\overline{\nabla}_{\mathcal{Z}}\varphi) \mathcal{X}\bigr) + \mu \upsilon(\mathcal{X}) \cos^{2} \vartheta_{2} \Vert \mathcal{Z} \Vert ^{2} \\ &{} + g\bigl(\zeta(\mathcal{X}, \mathcal{Z}), \mathcal{F}\mathcal{P}\mathcal{Z} \bigr). \end{aligned} $$

Again using (2), we derive $g(\mathcal{P}\mathcal{Z}, (\overline{\nabla}_{\mathcal{Z}}\varphi)\mathcal{X}) = g(\mathcal{X}, (\overline{\nabla}_{\mathcal{P}\mathcal{Z}}\varphi)\mathcal{Z})$. For $\mathcal{X}, \mathcal{Y} \in \Gamma(T\mathcal{M}_{1})$ and $\mathcal{Z} \in \Gamma(T\mathcal{M}_{2})$, we have $g(\nabla_{\mathcal{P}\mathcal{Z}} \varphi \mathcal{X}, \mathcal{Z}) = g(\overline{\nabla}_{\mathcal{P}\mathcal{Z}}\varphi \mathcal{X}, \mathcal{Z})$. This gives

8 $$ \begin{aligned}[b] 0 &= (\varphi \mathcal{X} \ln h)g(\mathcal{P} \mathcal{Z}, \mathcal{Z}) = g(\mathcal{X}, \varphi \overline{\nabla}_{\mathcal{P}\mathcal{Z}} \mathcal{Z}) \\ &= g(\mathcal{X}, \overline{\nabla}_{\mathcal{P}\mathcal{Z}} \mathcal{P}\mathcal{Z}) + g( \mathcal{X}, \overline{\nabla}_{\mathcal{P}\mathcal{Z}} \mathcal{F}\mathcal{Z}) - g\bigl( \mathcal{X}, (\overline{\nabla}_{\mathcal{P}\mathcal{Z}}\varphi)\mathcal{Z}\bigr) \\ &= - g(\nabla_{\mathcal{P}\mathcal{Z}}\mathcal{X}, \mathcal{P}\mathcal{Z}) - g( \mathcal{X}, \Lambda_{\mathcal{F}\mathcal{Z}}\mathcal{P}\mathcal{Z}) - g\bigl(\mathcal{P} \mathcal{Z}, (\overline{\nabla}_{\mathcal{Z}}\varphi)\mathcal{X}\bigr), \end{aligned} $$

or

$$g\bigl(\mathcal{P}\mathcal{Z}, (\overline{ \nabla}_{\mathcal{Z}}\varphi)\mathcal{X}\bigr) = - (\mathcal{X} \ln h) \cos^{2}\vartheta_{2} \Vert \mathcal{Z} \Vert ^{2} - g\bigl(\zeta(\mathcal{X}, \mathcal{P}\mathcal{Z}), \mathcal{F} \mathcal{Z}\bigr), $$

where we have used (3), (2), and Lemma 1$(ii)$. Plugging (8) into (7), we have the following:

9 $$ \begin{aligned}[b] 2 g\bigl(\zeta(\mathcal{X}, \mathcal{P} \mathcal{Z}), \mathcal{F}\mathcal{Z}\bigr) ={}& - (\mathcal{X} \ln h) \cos^{2}\vartheta_{2} \Vert \mathcal{Z} \Vert ^{2} + \mu \upsilon(\mathcal{X}) \cos^{2} \vartheta_{2} \Vert \mathcal{Z} \Vert ^{2} \\ &{} + g\bigl(\zeta(\mathcal{X}, \mathcal{Z}), \mathcal{F}\mathcal{P}\mathcal{Z} \bigr). \end{aligned} $$

Also, we deduce

10 $$ \begin{aligned}[b] g\bigl(\mathcal{P}\mathcal{Z}, (\overline{ \nabla}_{\mathcal{X}}\varphi)\mathcal{Z}\bigr) ={}& (\mathcal{X} \ln h) \cos^{2}\vartheta_{2} \Vert \mathcal{Z} \Vert ^{2} + g\bigl(\zeta(\mathcal{X}, \mathcal{P}\mathcal{Z}), \mathcal{F} \mathcal{Z}\bigr) \\ &{} - \mu \upsilon(\mathcal{X}) \cos^{2}\vartheta_{2} \Vert \mathcal{Z} \Vert ^{2}. \end{aligned} $$

Replacing $\mathcal{Z}$ by $\mathcal{P}\mathcal{Z}$ in (10), we get

11 $$ \begin{aligned}[b] g\bigl(\mathcal{P}\mathcal{Z}, (\overline{ \nabla}_{\mathcal{X}}\varphi)\mathcal{Z}\bigr) ={}& (\mathcal{X} \ln h) \cos^{2}\vartheta_{2} \Vert \mathcal{Z} \Vert ^{2} - g\bigl(\zeta(\mathcal{X}, \mathcal{Z}), \mathcal{F}\mathcal{P} \mathcal{Z}\bigr) \\ &{} - \mu \upsilon(\mathcal{X}) \cos^{2}\vartheta_{2} \Vert \mathcal{Z} \Vert ^{2}. \end{aligned} $$

Combining equations (10) and (11), we arrive at

12 $$\begin{aligned} g\bigl(\zeta(\mathcal{X}, \mathcal{P}\mathcal{Z}), \mathcal{F} \mathcal{Z}\bigr) = - g\bigl(\zeta(\mathcal{X}, \mathcal{Z}), \mathcal{F} \mathcal{P}\mathcal{Z}\bigr). \end{aligned}$$

Hence, (9) and (12) give assertion $(vi)$. This completes the proof of our lemma. □

## Bounds for the squared norm of the second fundamental form

Let $\mathcal{M} = \mathcal{M}_{1} \times_{h} \mathcal{M}_{2}$ be a warped product bi-slant submanifold of a nearly trans-Sasakian manifold $(\overline{\mathcal{M}}, \varphi, \xi, \upsilon, g)$, where $\mathcal{M}_{1}$ and $\mathcal{M}_{2}$ are proper slant submanifolds with slant angles $\vartheta_{1}$ and $\vartheta_{2}$, respectively. Further, we assume that $\operatorname{dim}(\overline{\mathcal{M}}) = 2n + 1$, $\operatorname{dim}(\mathcal{M}_{1}) = 2a + 1$, $\operatorname{dim}(\mathcal{M}_{2}) = 2b$, and $\operatorname{dim}(\mathcal{M}) = m = 2a + 2b + 1$. Let $\mathcal{D}_{1}$ and $\mathcal{D}_{2}$ be the tangent bundles on $\mathcal{M}_{1}$ and $\mathcal{M}_{2}$, respectively. We assume that [19]

(i) $\{\mathcal{E}_{1}, \dots, \mathcal{E}_{a}, \mathcal{E}_{a + 1} = \sec \vartheta_{1} \mathcal{P}\mathcal{E}_{1}, \dots, \mathcal{E}_{2a} = \sec \vartheta_{1} \mathcal{P}\mathcal{E}_{a}, \mathcal{E}_{2a + 1} = \xi\}$ is a local orthonormal frame of $\mathcal{D}_{1}$.
(ii) $\{\mathcal{E}_{2a + 2} = \mathcal{E}^{*}_{1}, \dots, \mathcal{E}_{2a + b + 1} = \mathcal{E}^{*}_{b}, \mathcal{E}_{2a + b + 2} = \mathcal{E}^{*}_{b + 1} = \sec \vartheta_{2} \mathcal{P}\mathcal{E}^{*}_{1}, \dots, \mathcal{E}_{m} = \mathcal{E}_{2a + 2b + 1} = \mathcal{E}^{*}_{2b} = \sec \vartheta_{2} \mathcal{P}\mathcal{E}^{*}_{b}\}$ is a local orthonormal frame of $\mathcal{D}_{2}$.
(iii) $\{\mathcal{E}_{m + 1} = \tilde{\mathcal{E}}_{1} = \csc \vartheta_{1} \mathcal{F}\mathcal{E}_{1}, \dots, \mathcal{E}_{m + a} = \tilde{\mathcal{E}}_{a} = \csc \vartheta_{1} \mathcal{F}\mathcal{E}_{a}, \mathcal{E}_{m + a + 1} = \tilde{\mathcal{E}}_{a + 1} = \csc \vartheta_{1} \sec \vartheta_{1} \mathcal{F}\mathcal{P}\mathcal{E}_{1}, \dots, \mathcal{E}_{m + 2a} = \tilde{\mathcal{E}}_{2a} = \csc \vartheta_{1} \sec \vartheta_{1} \mathcal{F}\mathcal{P}\mathcal{E}_{a}\}$ is a local orthonormal frame of $\mathcal{F}\mathcal{D}_{1}$.
(iv) $\{\mathcal{E}_{m + 2a + 1} = \tilde{\mathcal{E}}_{1} = \csc \vartheta_{2} \mathcal{F}\mathcal{E}^{*}_{1}, \dots, \mathcal{E}_{m + 2a + b} = \tilde{\mathcal{E}}_{b} = \csc \vartheta_{2} \mathcal{F}\mathcal{E}^{*}_{b}, \mathcal{E}_{m + 2a + b + 1} = \tilde{\mathcal{E}}_{b + 1} = \csc \vartheta_{2} \sec \vartheta_{2} \mathcal{F}\mathcal{P}\mathcal{E}^{*}_{1}, \dots, \mathcal{E}_{2m - 1} = \tilde{\mathcal{E}}_{2b} = \csc \vartheta_{2} \sec \vartheta_{2} \mathcal{F}\mathcal{P}\mathcal{E}^{*}_{b}\}$ is a local orthonormal frame of $\mathcal{F}\mathcal{D}_{2}$.
(v) $\{\mathcal{E}_{2m}, \dots, \mathcal{E}_{2n + 1}\}$ is a local orthonormal frame of *ν*.

Now, we prove our main theorem of this paper.

### Theorem 1

*Let* $\mathcal{M} = \mathcal{M}_{1} \times_{h} \mathcal{M}_{2}$ *be a warped product bi*-*slant submanifold of a nearly trans*-*Sasakian manifold* $(\overline{\mathcal{M}}, \varphi, \xi, \upsilon, g)$ *such that* $\mathcal{M}_{1}$ *and* $\mathcal{M}_{2}$ *are proper slant submanifolds with slant angles* $\vartheta_{1}$ *and* $\vartheta_{2}$, *respectively*. *If* $\mathcal{M}$ *is* $\mathcal{D}_{2}$-*totally geodesic*, *then we have the following*:

(i) *The squared norm of the second fundamental form* *ζ* *of* $\mathcal{M}$ *satisfies*
  13 $$ \Vert \zeta \Vert ^{2} \geq 4 b \csc^{2} \vartheta_{2} \biggl[\biggl(\cos^{2} \vartheta_{1} + \frac{1}{9} \cos^{2} \vartheta_{2}\biggr) \bigl( \Vert \nabla \ln h \Vert ^{2} - \mu^{2}\bigr) + \lambda^{2} \biggr]. $$
  *Furthermore*,
  - *For a nearly Sasakian manifold* $(\overline{\mathcal{M}}, \varphi, \xi, \upsilon, g)$, *ζ* *of* $\mathcal{M}$ *satisfies*
    14 $$ \Vert \zeta \Vert ^{2} \geq 4b \csc^{2} \vartheta_{2} \biggl[\biggl(\cos^{2} \vartheta_{1} + \frac{1}{9} \cos^{2} \vartheta_{2}\biggr) \bigl( \Vert \nabla \ln h \Vert ^{2}\bigr) + 1 \biggr]. $$
  - *For a nearly Kenmotsu manifold* $(\overline{\mathcal{M}}, \varphi, \xi, \upsilon, g)$, *ζ* *of* $\mathcal{M}$ *satisfies*
    15 $$ \Vert \zeta \Vert ^{2} \geq 4b \csc^{2} \vartheta_{2} \biggl(\cos^{2} \vartheta_{1} + \frac{1}{9} \cos^{2} \vartheta_{2}\biggr) \bigl( \Vert \nabla \ln h \Vert ^{2} - 1\bigr). $$
  - *For a nearly cosymplectic manifold* $(\overline{\mathcal{M}}, \varphi, \xi, \upsilon, g)$, *ζ* *of* $\mathcal{M}$ *satisfies*
    16 $$ \Vert \zeta \Vert ^{2} \geq 4b \csc^{2} \vartheta_{2}\biggl(\cos^{2} \vartheta_{1} + \frac{1}{9} \cos^{2} \vartheta_{2}\biggr) \bigl( \Vert \nabla \ln h \Vert ^{2}\bigr). $$
(ii) *If the equality sign holds in all four cases*, *then* $\mathcal{M}_{1}$ *is a totally geodesic submanifold of* $\overline{\mathcal{M}}$ *and* $\mathcal{M}_{2}$ *is a totally umbilical submanifold of* $\overline{\mathcal{M}}$. *In other words*, $\mathcal{M}$ *is a minimal submanifold of* $\overline{\mathcal{M}}$.

### Proof

The squared norm of the second fundamental form *ζ* is defined by

$$\begin{aligned} \Vert \zeta \Vert ^{2} =& \sum_{i,j = 1}^{m}g \bigl(\zeta(\mathcal{E}_{i}, \mathcal{E}_{j}), \zeta( \mathcal{E}_{i}, \mathcal{E}_{j})\bigr) = \sum _{r = m + 1}^{2n + 1}\sum_{i,j = 1}^{m}g \bigl(\zeta(\mathcal{E}_{i}, \mathcal{E}_{j}), \mathcal{E}_{r}\bigr)^{2}. \end{aligned}$$

From the assumed frames, the above equation can be written as

17 $$ \begin{aligned}[b] \Vert \zeta \Vert ^{2} ={}& \sum _{r = m + 1}^{2n + 1}\sum_{i,j = 1}^{2a + 1}g \bigl(\zeta(\mathcal{E}_{i}, \mathcal{E}_{j}), \mathcal{E}_{r}\bigr)^{2} + 2\sum _{r = m + 1}^{2n + 1}\sum_{i = 1}^{2a + 1} \sum_{j = 1}^{2b}g\bigl(\zeta\bigl( \mathcal{E}_{i}, \mathcal{E}^{*}_{j}\bigr), \mathcal{E}_{r}\bigr)^{2} \\ &{} + \sum_{r = m + 1}^{2n + 1}\sum _{i,j = 1}^{2b}g\bigl(\zeta\bigl(\mathcal{E}^{*}_{i}, \mathcal{E}^{*}_{j}\bigr), \mathcal{E}_{r} \bigr)^{2}. \end{aligned} $$

Using the hypothesis and leaving the first term on the right-hand side of (17) to introduce the inequality, we obtain

$$\begin{aligned} \Vert \zeta \Vert ^{2} \geq 2\sum_{r = m + 1}^{2n + 1} \sum_{i = 1}^{2a + 1}\sum _{j = 1}^{2b}g\bigl(\zeta\bigl(\mathcal{E}_{i}, \mathcal{E}^{*}_{j}\bigr), \mathcal{E}_{r} \bigr)^{2}. \end{aligned}$$

Decomposing the above equation according to (5), we derive

18 $$ \begin{aligned}[b] \Vert \zeta \Vert ^{2}\geq{}& 2 \Biggl[\sum_{r = m + 1}^{2a + m}\sum _{i = 1}^{2a + 1}\sum_{j = 1}^{2b}g \bigl(\zeta\bigl(\mathcal{E}_{i}, \mathcal{E}^{*}_{j} \bigr), \tilde{\mathcal{E}}_{r}\bigr)^{2} \\ &{} + \sum_{r = 2a + m + 1}^{2b + 2a + m}\sum _{i = 1}^{2a + 1}\sum_{j = 1}^{2b}g \bigl(\zeta\bigl(\mathcal{E}_{i}, \mathcal{E}^{*}_{j} \bigr), \tilde{\mathcal{E}}_{r}\bigr)^{2} + \sum _{r = 2m}^{2n + 1}\sum_{i = 1}^{2a + 1} \sum_{j = 1}^{2b}g\bigl(\zeta\bigl( \mathcal{E}_{i}, \mathcal{E}^{*}_{j}\bigr), \tilde{\mathcal{E}}_{r}\bigr)^{2} \Biggr]. \end{aligned} $$

Removing all the terms except for $\mathcal{F}\mathcal{D}_{2}$-components, we arrive at

19 $$ \begin{aligned} \Vert \zeta \Vert ^{2} &\geq 2 \Biggl(\sum_{r = 2a + m + 1}^{2b + 2a + m}\sum _{i = 1}^{2a + 1}\sum_{j = 1}^{2b}g \bigl(\zeta\bigl(\mathcal{E}_{i}, \mathcal{E}^{*}_{j} \bigr), \tilde{\mathcal{E}}_{r}\bigr)^{2} \Biggr), \\ \mbox{or}\quad \Vert \zeta \Vert ^{2} &\geq 2 \Biggl( \sum_{r = 1}^{2b}\sum _{i = 1}^{2a + 1}\sum_{j = 1}^{2b}g \bigl(\zeta\bigl(\mathcal{E}_{i}, \mathcal{E}^{*}_{j} \bigr), \tilde{\mathcal{E}}_{r}\bigr)^{2} \Biggr). \end{aligned} $$

Thus, by using the orthonormal frame fields of $\mathcal{D}_{1}, \mathcal{D}_{2}$ and $\mathcal{F}\mathcal{D}_{2}$, the above inequality reduces to

$$\begin{aligned} \Vert \zeta \Vert ^{2}\geq{}& 2 \Biggl[ \csc^{2} \vartheta_{2} \sum_{i = 1}^{a} \sum_{j = 1}^{b}g\bigl(\zeta\bigl( \mathcal{E}_{i}, \mathcal{E}^{*}_{j}\bigr), \mathcal{F}\mathcal{E}^{*}_{j}\bigr)^{2} \\ &{} + \csc^{2} \vartheta_{2} \sec^{2} \vartheta_{2} \sum_{i = 1}^{a}\sum _{j = 1}^{b}g\bigl(\zeta\bigl( \mathcal{E}_{i}, \mathcal{E}^{*}_{j}\bigr), \mathcal{F}\mathcal{P}\mathcal{E}^{*}_{j}\bigr)^{2} \\ &{} + \csc^{2} \vartheta_{2} \sec^{2} \vartheta_{2} \sum_{i = 1}^{a}\sum _{j = 1}^{b}g\bigl(\zeta\bigl( \mathcal{E}_{i}, \mathcal{P}\mathcal{E}^{*}_{j} \bigr), \mathcal{F}\mathcal{E}^{*}_{j}\bigr)^{2} \\ &{} + \csc^{2} \vartheta_{2} \sec^{2} \vartheta_{1} \sum_{i = 1}^{a}\sum _{j = 1}^{b}g\bigl(\zeta\bigl(P \mathcal{E}_{i}, \mathcal{E}^{*}_{j}\bigr), \mathcal{F}\mathcal{E}^{*}_{j}\bigr)^{2} \\ &{} + \csc^{2} \vartheta_{2} \sec^{2} \vartheta_{1} \sec^{2} \vartheta_{2} \sum _{i = 1}^{a}\sum_{j = 1}^{b}g \bigl(\zeta\bigl(\mathcal{P}\mathcal{E}_{i}, \mathcal{P} \mathcal{E}^{*}_{j}\bigr), \mathcal{F}\mathcal{E}^{*}_{j} \bigr)^{2} \\ &{} + \csc^{2} \vartheta_{2} \sec^{2} \vartheta_{1} \sec^{2} \vartheta_{2} \sum _{i = 1}^{a}\sum_{j = 1}^{b}g \bigl(\zeta\bigl(\mathcal{P}\mathcal{E}_{i}, \mathcal{E}^{*}_{j} \bigr), \mathcal{F}\mathcal{P}\mathcal{E}^{*}_{j} \bigr)^{2} \\ &{} + \csc^{2} \vartheta_{2} \sec^{4} \vartheta_{2} \sum_{i = 1}^{a}\sum _{j = 1}^{b}g\bigl(\zeta\bigl( \mathcal{E}_{i}, \mathcal{P}\mathcal{E}^{*}_{j} \bigr), \mathcal{F}\mathcal{P}\mathcal{E}^{*}_{j} \bigr)^{2} \\ &{} + \csc^{2} \vartheta_{2} \sec^{2} \vartheta_{1} \sec^{4} \vartheta_{2} \sum _{i = 1}^{a}\sum_{j = 1}^{b}g \bigl(\zeta\bigl(\mathcal{P}\mathcal{E}_{i}, \mathcal{P} \mathcal{E}^{*}_{j}\bigr), \mathcal{F}\mathcal{P} \mathcal{E}^{*}_{j}\bigr)^{2} \\ &{} + \csc^{2} \vartheta_{2} \sum _{j = 1}^{b}g\bigl(\zeta\bigl(\xi, \mathcal{E}^{*}_{j}\bigr), \mathcal{F}\mathcal{E}^{*}_{j} \bigr)^{2} \\ &{} + \csc^{2} \vartheta_{2} \sec^{2} \vartheta_{2} \sum_{j = 1}^{b}g \bigl(\zeta\bigl(\xi, \mathcal{E}^{*}_{j}\bigr), \mathcal{F} \mathcal{P}\mathcal{E}^{*}_{j}\bigr)^{2} \\ &{} + \csc^{2} \vartheta_{2} \sec^{2} \vartheta_{2} \sum_{j = 1}^{b}g \bigl(\zeta\bigl(\xi, P\mathcal{E}^{*}_{j}\bigr), \mathcal{F} \mathcal{E}^{*}_{j}\bigr)^{2} \\ &{} + \csc^{2} \vartheta_{2} \sec^{4} \vartheta_{2} \sum_{j = 1}^{b}g \bigl(\zeta\bigl(\xi, P\mathcal{E}^{*}_{j}\bigr), \mathcal{F} \mathcal{P}\mathcal{E}^{*}_{j}\bigr)^{2} \Biggr]. \end{aligned}$$

Using Lemma 2, the hypothesis, and the fact that

$$\begin{aligned} \Vert \nabla \ln h \Vert ^{2} = \sum_{i = 1}^{a} (\mathcal{E}_{i} \ln h)^{2} + \sum _{i = 1}^{a} \sec^{2} \vartheta_{1} (\mathcal{P}\mathcal{E}_{i} \ln h)^{2} + (\xi \ln h), \end{aligned}$$

we derive

$$ \begin{aligned} \Vert \zeta \Vert ^{2}\geq {}&4b \csc^{2} \vartheta_{2}\biggl(\cos^{2} \vartheta_{1} + \frac{1}{9} \cos^{2} \vartheta_{2}\biggr) \sum^{a}_{i = 1} \bigl[ \Vert \nabla \ln h \Vert ^{2} - 2\mu \upsilon( \mathcal{E}_{i}) (\mathcal{E}_{i} \ln h) \bigr] \\ &~{} + 4 b \csc^{2} \vartheta_{2} \lambda\Biggl(\lambda + \sum^{a}_{i = 1}\upsilon(\mathcal{E}_{i}) (\mathcal{P}\mathcal{E}_{i} \ln h)\Biggr). \end{aligned} $$

In view of the assumed orthonormal frame, the 1-form $\upsilon(\mathcal{E}_{i})$ is identically zero for all $i \in \{1, \dots, 2a\}$, the above expression can be modified as

$$\begin{aligned} \Vert \zeta \Vert ^{2} \geq& 4 b \csc^{2} \vartheta_{2} \biggl[\biggl(\cos^{2} \vartheta_{1} + \frac{1}{9} \cos^{2} \vartheta_{2}\biggr) \bigl( \Vert \nabla \ln h \Vert ^{2} - \mu^{2}\bigr) + \lambda^{2} \biggr]. \end{aligned}$$

This is the required inequality (i). Now, we discuss the following cases:

- For $\lambda = 1$ and $\mu = 0$, we have
  $$\begin{aligned} \Vert \zeta \Vert ^{2} \geq& 4b \csc^{2} \vartheta_{2} \biggl[\biggl(\cos^{2} \vartheta_{1} + \frac{1}{9} \cos^{2} \vartheta_{2}\biggr) \bigl( \Vert \nabla \ln h \Vert ^{2}\bigr) + 1 \biggr]. \end{aligned}$$
- For $\lambda = 0$ and $\mu = 1$, we have
  $$\begin{aligned} \Vert \zeta \Vert ^{2} \geq& 4b \csc^{2} \vartheta_{2} \biggl(\cos^{2} \vartheta_{1} + \frac{1}{9} \cos^{2} \vartheta_{2}\biggr) \bigl( \Vert \nabla \ln h \Vert ^{2} - 1\bigr). \end{aligned}$$
- For $\lambda = 0$ and $\mu = 0$, we have
  $$\begin{aligned} \Vert \zeta \Vert ^{2} \geq& 4b \csc^{2} \vartheta_{2} \biggl(\cos^{2} \vartheta_{1} + \frac{1}{9} \cos^{2} \vartheta_{2}\biggr) \bigl( \Vert \nabla \ln h \Vert ^{2}\bigr). \end{aligned}$$

If the equality holds in (13), then from (17) and the hypothesis of the theorem, we find that

20 $$\begin{aligned} \zeta(\mathcal{D}_{1}, \mathcal{D}_{1}) =& 0 \end{aligned}$$

and

21 $$\begin{aligned} \zeta(\mathcal{D}_{2}, \mathcal{D}_{2}) =& 0. \end{aligned}$$

Similarly, from (18), we get $\zeta(\mathcal{D}_{1}, \mathcal{D}_{2}) \perp \mathcal{F}\mathcal{D}_{1}$ and $\zeta(\mathcal{D}_{1}, \mathcal{D}_{2}) \perp \nu$, which further give

22 $$\begin{aligned} \zeta(\mathcal{D}_{1}, \mathcal{D}_{2}) \subset& \mathcal{F}\mathcal{D}_{2}. \end{aligned}$$

Let $\zeta_{2}$ be the second fundamental form of $\mathcal{M}_{2}$ in $\mathcal{M}$, then for any $\mathcal{X} \in \Gamma(\mathcal{D}_{1})$ and $\mathcal{Z}, \mathcal{W} \in \Gamma(\mathcal{D}_{2})$, we have $g(\zeta_{2}(\mathcal{Z}, \mathcal{W}), \mathcal{X}) = g(\nabla_{\mathcal{Z}}\mathcal{W}, \mathcal{X}) = - g(\mathcal{W}, \nabla_{\mathcal{Z}}\mathcal{X})$. Thus, we derive from Lemma 1(ii) and (6) that $g(\zeta_{2}(\mathcal{Z}, \mathcal{W}), \mathcal{X}) = - g(\nabla h, \mathcal{X}) g(\mathcal{Z}, \mathcal{W})$, or

23 $$\begin{aligned} \zeta_{2}(\mathcal{Z}, \mathcal{W}) =& - \nabla h g( \mathcal{Z}, \mathcal{W}). \end{aligned}$$

By (20) and Lemma 1(i) ($\mathcal{M}_{1}$ is totally geodesic in $\mathcal{M}$), we conclude that $\mathcal{M}_{1}$ is totally geodesic in $\overline{\mathcal{M}}$. On the other hand, both (21) and (23) say that $\mathcal{M}_{2}$ is totally umbilical in $\overline{\mathcal{M}}$. Furthermore, relations (20), (21) and (22) imply that $\mathcal{M}$ is a minimal submanifold of $\overline{\mathcal{M}}$. Hence, our assertion (ii) is proved. □

### Remark 4

The purpose of taking $\zeta(\mathcal{D}_{2}, \mathcal{D}_{2}) = 0$ in Theorem 1 is to maintain the inequality and to get the required result when the equality holds in the derived inequality. One can obtain another inequality in Theorem 1 by assuming $\mathcal{M}$ is mixed totally geodesic.

## Some geometric applications and examples

The Hamiltonian *H* in a local orthonormal frame at a point ℘ is defined by

24 $$\begin{aligned} H(\nabla h, \wp) = \frac{1}{2}\sum _{j = 1}^{n}\bigl(dh(\mathcal{E}_{j}) \bigr)^{2} = \frac{1}{2} \sum_{j = 1}^{n} \bigl(\mathcal{E}_{j}(h)\bigr)^{2} = \frac{1}{2} \Vert \nabla h \Vert ^{2}. \end{aligned}$$

An optimal inequality (13) in terms of the Hamiltonian of a warping function ln*h* at a point $\wp \in \mathcal{M}$ takes the following form:

$$\begin{aligned} \Vert \zeta \Vert ^{2} \geq& 4 b \csc^{2} \vartheta_{2} \biggl[\biggl(\cos^{2} \vartheta_{1} + \frac{1}{9} \cos^{2} \vartheta_{2}\biggr) \bigl(2 H( \nabla \ln h, \wp) - \mu^{2}\bigr) + \lambda^{2} \biggr], \end{aligned}$$

where we have used (24). Similarly, we can easily find inequalities (14), (15), and (16) in terms of the Hamiltonian of a warping function at a point ℘.

In the case of inequality, we prove the triviality of warped product bi-slant submanifolds, which is as follows.

### Theorem 2

*On a compact oriented warped product bi*-*slant submanifold* $\mathcal{M} = \mathcal{M}_{1} \times_{h} \mathcal{M}_{2}$ *of a nearly trans*-*Sasakian manifold* $(\overline{\mathcal{M}}, \varphi, \xi, \upsilon, g)$. *If the following inequality*

25 $$\begin{aligned} \Vert \zeta \Vert ^{2} \leq& 4b \lambda^{2} \csc^{2}\vartheta_{2}- \mu^{2} \biggl(4 b \csc^{2} \vartheta_{2} \biggl(\cos^{2} \vartheta_{1} + \frac{1}{9} \cos^{2} \vartheta_{2}\biggr) \biggr) \end{aligned}$$

*holds*, *then* $\mathcal{M}$ *is a Riemannian product manifold*.

### Proof

Suppose that the inequality holds in Theorem 1(i), we obtain

$$\begin{aligned} \frac{ \Vert \zeta \Vert ^{2} - 4b \lambda^{2} \csc^{2}\vartheta_{2}}{4 b \csc^{2} \vartheta_{2} (\cos^{2} \vartheta_{1} + \frac{1}{9} \cos^{2} \vartheta_{2})} + \mu^{2} \geq& \bigl\Vert \nabla \ln h \bigr\Vert ^{2}. \end{aligned}$$

From the integration theory on manifolds, we derive

$$ \int_{\mathcal{M}} \Vert \nabla \ln h \Vert ^{2} \,dV \leq \int_{\mathcal{M}} \biggl(\frac{ \Vert \zeta \Vert ^{2} - 4b \lambda^{2} \csc^{2}\vartheta_{2}}{4 b \csc^{2} \vartheta_{2} (\cos^{2} \vartheta_{1} + \frac{1}{9} \cos^{2} \vartheta_{2})} + \mu^{2} \biggr) \,dV. $$

On account of hypothesis (25), we find that $\int_{\mathcal{M}} \|\nabla \ln h\|^{2} \,dV \leq 0$. From this, we say that $\|\nabla \ln h\|^{2} \leq 0$, but $0 \leq \|\nabla \ln h\|^{2}$, which further implies that $\|\nabla \ln h\| = 0$, that is, *h* is a constant function on $\mathcal{M}$. Thus, $\mathcal{M}$ is a Riemannian product manifold of two proper slant submanifolds $\mathcal{M}_{1}$ and $\mathcal{M}_{2}$ with slant angles $\vartheta_{1}$ and $\vartheta_{2}$, respectively. This completes the proof of the theorem. □

We provide some non-trivial examples of nearly trans-Sasakian manifolds and their warped product bi-slant submanifolds.

### Example 1

Consider a 7-dimensional differentiable manifold

$$\begin{aligned} \overline{\mathcal{M}} = \bigl\{ (x_{1}, y_{1}, x_{2}, y_{2}, x_{3}, y_{3}, z) \in \mathbb{R}^{7} | z \neq 0\bigr\} . \end{aligned}$$

We choose the vector fields

$$\begin{aligned} &\mathcal{E}_{1} = \mathrm{e}^{-z} \frac{\partial}{\partial x_{1}}, \qquad \mathcal{E}_{2} = \mathrm{e}^{-z} \frac{\partial}{\partial y_{1}}, \qquad \mathcal{E}_{3} = \mathrm{e}^{-z} \frac{\partial}{\partial x_{2}}, \qquad \mathcal{E}_{4} = \mathrm{e}^{-z} \frac{\partial}{\partial y_{2}}, \\ &\mathcal{E}_{5} = \mathrm{e}^{-z} \frac{\partial}{\partial x_{3}}, \qquad \mathcal{E}_{6} = \mathrm{e}^{-z} \frac{\partial}{\partial y_{3}}, \qquad \mathcal{E}_{7} = \xi = \frac{\partial}{\partial z}. \end{aligned}$$

Let *g* be the Riemannian metric defined by

$$\begin{aligned} g = \mathrm{e}^{2z}\bigl(dx_{1}^{2} + dy_{1}^{2} + dx_{2}^{2} + dx_{y}^{2} + dx_{3}^{2} + dy_{3}^{2}\bigr) + dz^{2}. \end{aligned}$$

Then we find that $g(\mathcal{E}_{i}, \mathcal{E}_{i}) = 1$ and $g(\mathcal{E}_{i}, \mathcal{E}_{j})$, ∀ $i, j = 1, \dots, 7$. Hence, $\{\mathcal{E}_{1}, \dots, \mathcal{E}_{7}\}$ is an orthonormal basis. Thus, the 1-form *υ* is defined by $\upsilon(\mathcal{X}) = g(\mathcal{X}, \xi)$ for any $\mathcal{X} \in \Gamma(T\overline{\mathcal{M}})$. Now, we define the $(1, 1)$-tensor field *φ* as

$$\begin{aligned} \varphi\biggl(\frac{\partial}{\partial x_{i}}\biggr) = \frac{\partial}{\partial y_{i}}, \qquad \varphi\biggl(\frac{\partial}{\partial y_{j}}\biggr) = - \frac{\partial}{\partial x_{j}}, \qquad \varphi\biggl(\frac{\partial}{\partial z}\biggr) = 0, \quad i, j = 1, \dots, 6. \end{aligned}$$

By using the linearity of *φ* and *g*, we have (1). Hence, $(\overline{\mathcal{M}}, \varphi, \xi, \upsilon, g)$ is an almost contact metric manifold.

Let ∇̅ be the Levi–Civita connection with respect to *g*. Then we have

$$\begin{aligned}{} [\mathcal{E}_{i}, \mathcal{E}_{j}] = 0, \qquad [ \mathcal{E}_{i}, \xi] = [\mathcal{E}_{i}, \mathcal{E}_{7}] = \mathcal{E}_{i}, \quad \forall i, j = 1, \dots, 6, i \neq j. \end{aligned}$$

By using the Koszul formula for *g*, we calculate

$$\begin{aligned} &\overline{\nabla}_{\mathcal{E}_{i}}\mathcal{E}_{i} = \mathcal{E}_{7} = \xi, \qquad \overline{\nabla}_{\mathcal{E}_{i}} \mathcal{E}_{j} = \overline{\nabla}_{\mathcal{E}_{j}}\mathcal{E}_{i} = 0, \qquad \overline{\nabla}_{\mathcal{E}_{i}}\mathcal{E}_{7} = \overline{\nabla}_{\mathcal{E}_{i}}\xi = \mathcal{E}_{i}, \qquad \overline{\nabla}_{\mathcal{E}_{7}}\mathcal{E}_{i} = \overline{ \nabla}_{\xi}\mathcal{E}_{i} = 0 \\ &\quad \forall i, j = 1, \dots, 6, i \neq j. \end{aligned}$$

For any $\mathcal{X}, \mathcal{Y} \in \Gamma(T\overline{\mathcal{M}})$, we have

$$\begin{aligned} &\mathcal{X} = p_{1}\mathcal{E}_{1} + p_{2} \mathcal{E}_{2} + p_{3}\mathcal{E}_{3} + p_{4}\mathcal{E}_{4} + p_{5} \mathcal{E}_{5} + p_{6}\mathcal{E}_{6} + p_{7}\xi, \\ &\mathcal{Y} = q_{1}\mathcal{E}_{1} + q_{2} \mathcal{E}_{2} + q_{3}\mathcal{E}_{3} + q_{4}\mathcal{E}_{4} + q_{5} \mathcal{E}_{5} + q_{6}\mathcal{E}_{6} + q_{7}\xi, \\ &\varphi \mathcal{X} = p_{1}\mathcal{E}_{2} - p_{2}\mathcal{E}_{1} + p_{3}\mathcal{E}_{4} - p_{4}\mathcal{E}_{3} + p_{5} \mathcal{E}_{6} - p_{6}\mathcal{E}_{5}, \\ &\varphi \mathcal{Y} = q_{1}\mathcal{E}_{2} - q_{2}\mathcal{E}_{1} + q_{3}\mathcal{E}_{4} - q_{4}\mathcal{E}_{3} + q_{5} \mathcal{E}_{6} - q_{6}\mathcal{E}_{5}, \end{aligned}$$

where $p_{i}, q_{i} \in \mathbb{R}$, $i = 1, \dots, 7$. Now, we check equation (2). For this, we calculate

$$\begin{aligned} (\overline{\nabla}_{\mathcal{X}} \varphi)\mathcal{Y} + ( \overline{\nabla}_{\mathcal{Y}} \varphi)\mathcal{X} ={}& \overline{ \nabla}_{\mathcal{X}} \varphi \mathcal{Y} - \varphi \overline{ \nabla}_{\mathcal{X}} \mathcal{Y} + \overline{\nabla}_{\mathcal{Y}} \varphi \mathcal{X} - \varphi \overline{\nabla}_{\mathcal{Y}} \mathcal{X} \\ ={}& (- p_{1}q_{2} + p_{2}q_{1} - p_{3}q_{4} + p_{4}q_{3} - p_{5}q_{6} + p_{6} q_{5}) \xi \\ &{} - q_{7} (p_{1}\mathcal{E}_{2} - p_{2}\mathcal{E}_{1} + p_{3}\mathcal{E}_{4} - p_{4}\mathcal{E}_{3} + p_{5} \mathcal{E}_{6} - p_{6}\mathcal{E}_{5}) \\ &{}- (- p_{1}q_{2} + p_{2}q_{1} - p_{3}q_{4} + p_{4}q_{3} - p_{5}q_{6} + p_{6} q_{5}) \xi \\ &{}- p_{7}(q_{1}\mathcal{E}_{2} - q_{2}\mathcal{E}_{1} + q_{3}\mathcal{E}_{4} - q_{4}\mathcal{E}_{3} + q_{5} \mathcal{E}_{6} - q_{6}\mathcal{E}_{5}) \\ ={}& {-} q_{7} (p_{1}\mathcal{E}_{2} - p_{2}\mathcal{E}_{1} + p_{3}\mathcal{E}_{4} - p_{4}\mathcal{E}_{3} + p_{5} \mathcal{E}_{6} - p_{6}\mathcal{E}_{5}) \\ &{} - p_{7}(q_{1}\mathcal{E}_{2} - q_{2}\mathcal{E}_{1} + q_{3}\mathcal{E}_{4} - q_{4}\mathcal{E}_{3} + q_{5} \mathcal{E}_{6} - q_{6}\mathcal{E}_{5}) \\ ={}& {-}\bigl(\upsilon(\mathcal{Y})\varphi \mathcal{X} + \upsilon(\mathcal{X}) \varphi \mathcal{Y}\bigr) \end{aligned}$$

for any $\mathcal{X}, \mathcal{Y} \in \Gamma(T\overline{\mathcal{M}})$. Hence, $(\overline{\mathcal{M}}, \varphi, \xi, \upsilon, g)$ is a nearly trans-Sasakian manifold of type $(0, 1)$ or a nearly Kenmotsu manifold (see Remark 1).

### Example 2

The best example of a nearly cosymplectic manifold defined on a non-Euclidean space is $\mathbb{S}^{5}$. Following Remark 1, we say that $\mathbb{S}^{5}$ is a nearly trans-Sasakian manifold of type $(0, 0)$.

### Example 3

Let $\mathbb{R}^{9}$ be the Euclidean 9-space equipped with the Euclidean metric tensor *g*, the real global coordinates $(x_{1}, y_{1}, x_{2}, y_{2}, x_{3}, y_{3}, x_{4}, y_{4}, z)$, and the canonical structure *φ* given by

$$\begin{aligned} \varphi\biggl(\frac{\partial}{\partial x_{i}}\biggr) = \frac{\partial}{\partial y_{i}}, \qquad \varphi\biggl(\frac{\partial}{\partial y_{j}}\biggr) = - \frac{\partial}{\partial x_{j}}, \qquad \varphi\biggl(\frac{\partial}{\partial z}\biggr) = 0, \quad i,j = 1, \dots, 4. \end{aligned}$$

We can easily prove that $(\varphi, \xi = \frac{\partial}{\partial z}, \upsilon = dz, g = \upsilon \otimes \upsilon + \mathrm{e}^{2z} \sum_{i = 1}^{4}{(dx_{i} \otimes dx_{i} + dy_{i} \otimes dy_{i})})$ is an almost contact metric structure on $\mathbb{R}^{9}$. Also, it can be verified that $\mathbb{R}^{9}$ is a nearly trans-Sasakian manifold of type $(0,1)$ (see Example 1). Now, we consider a submanifold $\mathcal{M}$ in $\mathbb{R}^{9}$ defined by the immersion $\mathfrak{f}$ as follows:

26 $$\begin{aligned} \mathfrak{f}(u, v, w, p, q) = (u, v \sin \alpha_{1}, 0, v \cos \alpha_{1}, w, p \sin \alpha_{2}, 0, p \cos \alpha_{2}, q) \end{aligned}$$

for any constants $\alpha_{1}$ and $\alpha_{2}$. We choose the tangent bundle of $\mathcal{M}$ spanned by

$$\begin{aligned} &\mathcal{E}_{1} = \frac{\partial}{\partial x_{1}}, \qquad \mathcal{E}_{2} = \sin \alpha_{1} \frac{\partial}{\partial y_{1}} + \cos \alpha_{1} \frac{\partial}{\partial y_{2}}, \qquad \mathcal{E}_{3} = \frac{\partial}{\partial x_{3}}, \\ &\mathcal{E}_{4} = \sin \alpha_{2}\frac{\partial}{\partial y_{3}} + \cos \alpha_{2} \frac{\partial}{\partial y_{4}}, \qquad \mathcal{E}_{5} = \frac{\partial}{\partial q}. \end{aligned}$$

Furthermore, we have

$$\begin{aligned} &\varphi \mathcal{E}_{1} = \frac{\partial}{\partial y_{1}}, \qquad \varphi \mathcal{E}_{2} = - \sin \alpha_{1} \frac{\partial}{\partial x_{1}} - \cos \alpha_{1} \frac{\partial}{\partial x_{2}}, \qquad \varphi \mathcal{E}_{3} = \frac{\partial}{\partial y_{3}}, \\ &\varphi \mathcal{E}_{4} = - \sin \alpha_{2} \frac{\partial}{\partial x_{3}} - \cos \alpha_{2} \frac{\partial}{\partial x_{4}}, \qquad \varphi \mathcal{E}_{5} = 0, \end{aligned}$$

and the distributions are defined by $\mathcal{D}_{\vartheta_{1}} = \operatorname{Span}\{\mathcal{E}_{1}, \mathcal{E}_{2}\}$ and $\mathcal{D}_{\vartheta_{2}} = \operatorname{Span}\{\mathcal{E}_{3}, \mathcal{E}_{4}\}$. Then it can be easily seen that $\mathcal{D}_{\vartheta_{1}}$ is $\vartheta_{1}$-slant with $\vartheta_{1} = \arccos(\sin \alpha_{1})$ and $\mathcal{D}_{\vartheta_{2}}$ is $\vartheta_{2}$-slant with $\vartheta_{2} = \arccos(\sin \alpha_{2})$. Also, $\mathcal{E}_{5} = \xi$ is tangent to $\mathcal{D}_{\vartheta_{1}}$. Hence, $\mathfrak{f}$ defines a proper 5-dimensional bi-slant submanifold $\mathcal{M}$ with bi-slant angles $\{\arccos(\sin \alpha_{1}), \arccos(\sin \alpha_{2})\}$ in $\mathbb{R}^{9}$. It is clear that the distributions $\mathcal{D}_{\vartheta_{1}}$ and $\mathcal{D}_{\vartheta_{2}}$ are integrable. Also, we notice that $\nabla_{\mathcal{E}_{i}}\mathcal{E}_{j} = 0$, ∀ $i, j = 1, \dots, 4$. From which we say that $\mathcal{D}_{\vartheta_{1}}$ and $\mathcal{D}_{\vartheta_{2}}$ are totally geodesic and hence minimal.

### Example 4

We consider any submanifold $\mathcal{M}$ in a nearly trans-Sasakian manifold $\mathbb{R}^{7}$ (see Example 1)

27 $$\begin{aligned} \mathfrak{f}(u, v, w, q) = (u \cos v, w \cos v, u \sin v, w \sin v, w - u, w + u, q). \end{aligned}$$

The tangent bundle of $\mathcal{M}$ is spanned by

$$\begin{aligned} &\mathcal{E}_{1} = \cos v \frac{\partial}{\partial x_{1}} + \sin v \frac{\partial}{\partial x_{2}} - \frac{\partial}{\partial x_{3}} + \frac{\partial}{\partial y_{3}}, \\ &\mathcal{E}_{2} = - u \sin v \frac{\partial}{\partial x_{1}} + u \cos v \frac{\partial}{\partial x_{2}} - w \sin v \frac{\partial}{\partial y_{1}} + w \cos v \frac{\partial}{\partial y_{2}}, \\ &\mathcal{E}_{3} = \frac{\partial}{\partial x_{3}} + \cos v \frac{\partial}{\partial y_{1}} + \sin v \frac{\partial}{\partial y_{2}} + \frac{\partial}{\partial y_{3}}, \\ &\mathcal{E}_{4} = \frac{\partial}{\partial q}. \end{aligned}$$

Furthermore, we have

$$\begin{aligned} &\varphi \mathcal{E}_{1} = \cos v \frac{\partial}{\partial y_{1}} + \sin v \frac{\partial}{\partial y_{2}} - \frac{\partial}{\partial y_{3}} - \frac{\partial}{\partial x_{3}}, \\ &\varphi \mathcal{E}_{2} = - u \sin v \frac{\partial}{\partial y_{1}} + u \cos v \frac{\partial}{\partial y_{2}} + w \sin v \frac{\partial}{\partial x_{1}} - w \cos v \frac{\partial}{\partial x_{2}}, \\ &\varphi \mathcal{E}_{3} = \frac{\partial}{\partial y_{3}} - \cos v \frac{\partial}{\partial x_{1}} - \sin v \frac{\partial}{\partial x_{2}} - \frac{\partial}{\partial x_{3}}, \\ &\varphi \mathcal{E}_{4} = 0. \end{aligned}$$

It is easy to check that $\varphi \mathcal{E}_{2}$ is orthogonal to $T\mathcal{M}$. Then the proper slant and anti-invariant distributions of $\mathcal{M}$ are respectively defined by $\mathcal{D}_{\vartheta} = \operatorname{Span}\{\mathcal{E}_{1}, \mathcal{E}_{3}\}$ with slant angle $\vartheta = \arccos(\frac{1}{3})$ and $\mathcal{D}_{\perp} = \operatorname{Span}\{\mathcal{E}_{2}\}$. Also, $\mathcal{E}_{4} = \xi$ is tangent to $\mathcal{D}_{\vartheta}$. Hence, $\mathfrak{f}$ defines a proper 4-dimensional pseudo-slant submanifold (bi-slant submanifold with bi-slant angles $\{\arccos(\frac{1}{3}), \frac{\pi}{2}\}$) $\mathcal{M}$ in $\mathbb{R}^{7}$. It is easy to check that the distributions $\mathcal{D}_{\vartheta}$ and $\mathcal{D}_{\perp}$ are integrable.

### Example 5

In the continuation of Example 3, we consider that $\mathcal{M}_{1}$ and $\mathcal{M}_{2}$ are respectively the integral manifolds of $\mathcal{D}_{\vartheta_{1}}$ and $\mathcal{D}_{\vartheta_{2}}$. Then it follows from Definition 5 and (26) that the induced metric tensor *g* of the product manifold $\mathcal{M}$ of $\mathcal{M}_{1}$ and $\mathcal{M}_{2}$ is given by

$$ \begin{aligned} g &= du^{2} + \bigl(\sin^{2} \alpha_{1} + \cos^{2}\alpha_{1}\bigr) dv^{2} + dw^{2} + \bigl(\sin^{2} \alpha_{2} + \cos^{2}\alpha_{2}\bigr) dp^{2} + dq^{2} \\ &= du^{2} + dv^{2} + dq^{2} + dw^{2} + dp^{2} \\ &= g_{1} + g_{2}, \end{aligned} $$

where $g_{1} = du^{2} + dv^{2} + dq^{2}$ and $g_{2} = dw^{2} + dp^{2}$ are respectively the metric tensors of $\mathcal{M}_{1}$ and $\mathcal{M}_{2}$. Consequently, $\mathcal{M} = \mathcal{M}_{1} \times_{h} \mathcal{M}_{2}$ is a warped product bi-slant submanifold of $\mathbb{R}^{9}$ with the constant warping function, i.e., $h = 1$ such that *ξ* is tangent to $\mathcal{M}_{1}$. Thus, $\mathcal{M}$ is a Riemannian product manifold.

### Example 6

In the continuation of Example 4, we assume that $\mathcal{M}_{1}$ and $\mathcal{M}_{2}$ are the integral manifolds of $\mathcal{D}_{\vartheta}$ and $\mathcal{D}_{\perp}$, respectively. Then it follows from Definition 5 and (27) that the induced metric tensor *g* of $\mathcal{M}$ is given by

$$ \begin{aligned} g ={}& \bigl(\cos^{2}v + \sin^{2}v + 2 \bigr)du^{2} + \bigl(u^{2}\sin^{2}v + u^{2}\cos^{2}v + w^{2} \sin^{2}v + w^{2} \cos^{2}v\bigr) dv^{2} \\ &{} + \bigl(\cos^{2}v + \sin^{2}v + 2\bigr)dw^{2} + dq^{2} \\ ={}& 3\bigl(du^{2} + dw^{2}\bigr) + dq^{2} + \bigl(u^{2} + w^{2}\bigr)dv^{2} = g_{1} + g_{2}, \end{aligned} $$

where $g_{1} = 3(du^{2} + dw^{2}) + dq^{2}$ and $g_{2} = (u^{2} + w^{2})dv^{2}$ are respectively the metric tensors of $\mathcal{M}_{1}$ and $\mathcal{M}_{2}$. As a consequence, $\mathcal{M} = \mathcal{M}_{1} \times_{h} \mathcal{M}_{2}$ is a warped product pseudo-slant submanifold of $\mathbb{R}^{7}$ with a warping function, i.e., $h = \sqrt{u^{2} + w^{2}}$ such that *ξ* is tangent to $\mathcal{M}_{1}$.
